# *Varibaculum cambriense* Infections in Hong Kong, China, 2006

**DOI:** 10.3201/eid1507.081291

**Published:** 2009-07

**Authors:** Yiu-Wai Chu, Chi-Ho Wong, Man-Yu Chu, Cara P.F. Cheung, Terence K.M. Cheung, Cindy Tse, Wei-Kwang Luk, Janice Y.C. Lo

**Affiliations:** Centre for Health Protection, Hong Kong, People’s Republic of China (Y.-W. Chu, C.-H. Wong, M.-Y. Chu, C.P.F. Cheung, T.K.M. Cheung, J.Y.C. Lo); Kwong Wah Hospital, Hong Kong (C. Tse); and Tseung Kwan O Hospital, Hong Kong (W.-K. Luk)

**Keywords:** Anaerobe, Varibaculum cambriense, soft tissue infections, 16S rRNA gene sequencing, bacteria, Hong Kong, letter

**To the Editor:** *Varibaculum cambriense* is an anaerobic, gram-positive, diphtheroid bacterium that was described by Hall et al. in 2003 (*1*). Biochemical testing, electrophoretic analysis of whole-cell proteins, and phylogenetic analysis of 16S rRNA gene sequences showed that *V*. *cambriense* is related to but distinct from *Actinomyces* spp. and related taxa, including the genera *Actinobaculum*, *Arcanobacterium*, and *Mobiluncus*.

Although its natural habitat remains unknown, *V*. *cambriense* has been isolated from intrauterine devices and human vagina and abscess specimens (*2*). Commercial systems, such as analytical profile index (API) Rapid ID 32 Strep and Coryne kits (bioMérieux, Marcy l’Etoile, France), used in differentiation of novel bacteria provide biochemical profiles useful for identification of *V*. *cambriense* (*1*). However, the absence of data on this organism in manufacturers’ databases has hampered recognition of *V*. *cambriense* in routine clinical laboratories. We report 4 cases of *V*. *cambriense* infection and show that this bacterium is a potential pathogen in skin and soft tissue infections.

In 2006, four isolates of gram-positive curved bacilli that grew on Columbia agar with 5% horse blood under anaerobic conditions were referred by 2 regional hospitals in Hong Kong to our laboratory for identification. These isolates originated from the abscess specimens of 4 patients.

Patient 1 was a 45-year-old woman with a right ovarian chocolate cyst and endometriosis who had undergone laparotomy, right salpingo-oophorectomy, and lysis of adhesions in 2001. Since then, she had a recurrent abscess over the umbilical scar that was treated conservatively. Culture of pus from the umbilical scar grew an unidentified gram-positive bacillus (M124). Histologic analysis of umbilical tissue showed acute suppurative inflammatory cells and microabscess formation. The patient refused follow-up and no antimicrobial drug treatment was given.

Patient 2 was a 25-year-old man who had a history of excision of multiple sebaceous cysts in the groin, and buttock pain and swelling for 4 days. He had no previous trauma and was afebrile. An abscess was incised and drained. Culture of tissue obtained grew an unidentified gram-positive bacillus (M397) and a *Peptostreptococcus* spp. The infection later subsided without antimicrobial drug treatment.

Patient 3 was a 34-year-old man with a lump in the left groin that had been present for 1 year. He was hospitalized with erythema and increased swelling of the lesion. An abscess was diagnosed, incised, and drained. Gram staining of pus showed numerous leukocytes and gram-positive bacilli. Culture yielded an unidentified gram-positive bacillus (M380). He was treated with ampicillin and cloxacillin, and the wound healed.

Patient 4 was a 55-year-old woman with an abscess on her back. She had a 3-year history of seronegative rheumatoid arthritis but was not receiving immunosuppressant therapy. The abscess was drained, and gram staining of pus showed numerous leukocytes and gram-positive cocci and bacilli. Culture yielded *Peptostreptococcus* spp. and an unidentified gram-positive bacillus (M398). The abscess healed without antimicrobial drug treatment.

The 4 unidentified gram-positive bacilli from these patients were initially characterized by using the API Rapid ID 32 Strep and Coryne kits. Doubtful identifications at various confidence levels were obtained, including *Corynebacterium diphtheriae* var. *mitis* or var. *belfanti*, *Gardnerella vaginalis*, *Streptococcus mitis*, *S*. *oralis*, *Gemella morbillorum*, and *Aerococcus urinae*. Sequencing of full length 16S rRNA genes suggested that the isolates were *V*. *cambriense*; GenBank BLAST (http://blast.ncbi.nlm.nih.gov/Blast.cgi) results showed *V*. *cambriense* type strain CCUG 44998^T^ sequence as the best match (identities 98%–99%) (*3*).

Biochemical reactions of the strains were similar to those of CCUG 44998^T^ (*1*). All 4 isolates grew poorly in air and 5% CO_2_, were catalase negative, did not hydrolyse esculin or gelatin, or reduce nitrates. The 4 isolates did not ferment most of the carbohydrates in the 2 API kits, except for ribose, maltose, glucose, and sucrose. Discrepancies in results between the 2 test kits were seen with ribose in 1 isolate (M398) and maltose in 2 isolates (M124 and M397). One isolate (M380) did not hydrolyze hippurate but produced acid from trehalose and xylose. This isolate was also α-galactosidase positive, a result different from that of the type strain. All 4 isolates were α-glucosidase positive and 3 were alanyl-phenyl-alanyl-proline arylamidase positive. Some of the biochemical reactions for the 4 isolates, including all tests for delineating *V*. *cambriense* from other catalase-negative *Actinomyces* spp. (*1*), are summarized in the Table.

**Table:** Characteristics and test results for 4 isolates of unknown bacteria, Hong Kong, China, 2006*

Characteristic	*Varibaculum cambriense* type strain CCUG 44998^T^	Isolate			
		M124	M397	M380	M398
Hospital		B	B	A	A
Patient sex/age, y		F/45	M/25	M/34	F/55
Date referred/isolated		2006 May	2006 Dec	2006 Dec	2006 Dec
API test code					
Rapid ID32 Strep		00100010100	00102010100	02122001100	00002011100
Coryne		1010321	1410321	1010721	1010321
Acid production from					
Glucose	+	+	+	+	+
Lactose	–	–	–	–	–
Maltose	V	– (+)	– (+)	+	+
Melibiose	–	–	–	–	–
Pullulan	–	–	–	–	–
Raffinose	–	–	–	–	–
Ribose	V	+	+	+	– (+)
Sucrose	V	+	+	+	+
Trehalose	V	–	–	+	–
MBDG	–	–	–	–	–
Hydrolysis of hippurate	V	+	+	–	+
Production of					
α-galactosidase	–	–	–	+	–
β-galactosidase	V	–	– (+)	–	–
α-glucosidase	+	– (+)	– (+)	– (+)	– (+)
Alkaline phosphatase	–	–	–	–	–
APPA	V	–	+	+	+
GenBank accession no.†		FJ169866	FJ169867	FJ169868	FJ169869
Identity to type strain CCUG 44998^T^, %		99	98	99	99

*API, analytical profile index; V, variable; MBDG, methyl-β-d-glucopyranoside; APPA, alanyl-phenylalanyl-proline arylamidase. Test results were obtained by using the API Rapid ID32 Strep kit (bioMérieux, Marcy l’Etoile, France). Results in parentheses were obtained by using the API Coryne kit (bioMérieux). †For 16S rRNA gene sequence.

We report the isolation of *V*. *cambriense* from 4 patients with purulent skin and soft tissue infections. Our findings contribute to understanding of the clinical and pathogenic potential of this anaerobic bacterium. Gram-positive diphtheroid organisms from wound specimens are occasionally considered to be skin commensal organisms. Clinical microbiologists should be aware of this organism and the current inadequacy of commercial systems for its identification. We have shown that 16S rRNA gene sequencing is a useful alternative to gas–liquid chromatographic analyses of cell wall fatty acids or metabolic products for identification of anaerobic gram-positive bacilli.
